# Trophic interrelationships of bacteria are important for shaping soil protist communities

**DOI:** 10.1111/1758-2229.13143

**Published:** 2023-03-29

**Authors:** Thi Bao Anh Nguyen, Qing‐Lin Chen, Zhen‐Zhen Yan, Chaoyu Li, Ji‐Zheng He, Hang‐Wei Hu

**Affiliations:** ^1^ School of Agriculture and Food, Faculty of Science University of Melbourne Parkville Victoria Australia

## Abstract

Protists occupy multiple trophic positions in soil food webs and significantly contribute to organic matter decomposition and biogeochemical cycling. Protists can ingest bacteria and fungi as main food sources while being subjected to predation of invertebrates, but our understanding of how bottom‐up and top‐down regulations structure protists in natural soil habitats is limited. Here, we disentangle the effects of trophic regulations to the diversity and structure of soil protists in natural settings across northern and eastern Australia. Bacterial and invertebrate diversity were identified as important drivers of the diversity of functional groups of protists. Moreover, the compositions of protistan taxonomic and functional groups were better predicted by bacteria and fungi, than by soil invertebrates. There were strong trophic interconnections between protists and bacteria in multiple organismic network analysis. Altogether, the study provided new evidence that, bottom‐up control of bacteria played an important role in shaping the soil protist community structure, which can be derived from feeding preferences of protists on microbial prey, and their intimate relationships in soil functioning or environmental adaptation. Our findings advance our knowledge about the impacts of different trophic groups on key soil organismic communities, with implications for ecosystem functions and services.

## INTRODUCTION

Soils are the most diverse habitat for organisms on Earth, harbouring approximately 25% of all living creatures (Bardgett & van der Putten, 2014; Fierer, 2017) and underpinning essential ecological functions and services (Fao et al., 2020). Soil organisms include archaea and bacteria, viruses, and various eukaryotes (protists, fungi, nematodes, and other fauna) residing in soils (Fierer, 2017). Soil organisms occupy various trophic levels in the soil food web and are shaped by both top‐down and bottom‐up controls (Leroux & Loreau, 2015). Top‐down control refers to the force that predators or consumers influence their prey, while bottom‐up control is the force of prey or nutrient resources regulating their predators or consumers. Bacteria and fungi, are functionally defined as the base of the soil food web, playing a critical role in soil functions (carbon transformation, soil structure formation, and nutrient cycling) (Fao et al., 2020). At higher trophic levels, protists and soil invertebrates (nematodes, arthropods, tardigrades, rotifera, etc.) act as important biological regulators of microbial prey bacteria and fungi. Despite the importance of trophic regulations, we lack an understanding of how these trophic regulations shape the diversity and community of soil organisms in terrestrial ecosystems (Thakur & Geisen, 2019).

Protists, pivotal, and dominant eukaryotes in soil ecosystems, are classified into different functional groups including consumers—important regulators of microbial communities; phototrophs—carbon fixers; and parasites of plants and animals. Protistan consumers feed wide‐spectrum organisms including bacteria, fungi, and other eukaryotes, contributing to nutrient cycling, antimicrobial resistance, and plant performance (Nguyen et al., 2020a; Xiong et al., 2020; Nguyen et al., 2022). In soils, bacteria and fungi are main nutrient sources of protists while protists are consumed by higher trophic‐level invertebrates (Geisen et al., 2018). A few previous studies have examined the effects of vegetation types, climate, edaphic and biotic factors (e.g., bacteria, fungi, and soil fauna) on the diversity and composition of soil protists at regional or global scales (Bates et al., 2013; Oliverio et al., 2020; Nguyen et al., 2021), but no studies have compared the relative importance of top‐down (invertebrates) and bottom‐up (microbial prey) controls in shaping the community structure of soil protists and their functional groups. A holistic understanding of the trophic relationships between protists and other organisms in the soil food web is pivotal to unravelling soil ecosystem processes and the alteration of soil biodiversity and functioning under global changes.

Although protists have a broad array of nutrients, they have a feeding preference on bacteria. In soil matrix, protists and bacteria share a habitat in similar‐size soil space and water‐filled microsites (Nielsen, 2019), hence they may frequently encounter and interact with each other. The protistan consumers, that is, primary controllers of bacteria and fungi, have also been reported as the most predominant group of the protist communities across regional and global soil ecosystems (Oliverio et al., 2020; Nguyen et al., 2021; Xiong et al., 2021). Notably, previous studies indicate that some protists (consumers or phototrophs) and bacteria or fungi may build the symbiotic relationships in mediating different ecosystem processes (e.g., biochemical cycle or bioremediation) and support each other to survive in severe environments (Gast et al., 2009; Ramanan et al., 2016; Subashchandrabose et al., 2013). Therefore, we hypothesized that the protist community would be more driven by bottom‐up control than by top‐down control. To test the hypothesis, we compared the relative importance of top‐down (soil invertebrates) and bottom‐up factors (bacteria and fungi) in regulating the protist diversity and community structure in 216 soil samples across Australia.

## EXPERIMENTAL PROCEDURES

### Soil sample collection

We collected a total of 216 soil samples (0–10 cm depth) from 72 sites over different vegetation types (native forests and woodlands, native shrublands, native grasslands, and plantation forests) in northern and eastern Australia. The sampling sites spanned from −19.2833° S to −38.1858° S and from 134.2° E to 153.6216° E (Figure S1). Three replicates were collected from a 20 × 20 m plot with minimal anthropogenic impacts at each site. The samples were kept on dry ice before arriving at the laboratory and sieved with 2 mm mesh to remove stones and plant roots. Soil samples were frozen at −20°C and used for molecular analyses to profile all tested soil organisms. The geographic information of the sampling sites and basic vegetation types, soil physicochemical properties (soil pH, total carbon, total nitrogen, nitrate nitrogen [NO_3_^−^–N], and ammonium nitrogen [NH_4_^+^–N]), and climatic factors (mean annual precipitation and mean annual temperature) is described in Table S1.

### Characterization of soil organisms

Total genomic DNA was extracted from 0.25 g of soil using a Power Soil DNA Isolation Kit (MO BIO laboratories, Inc.) according to the manufacturer's instruction. Soil protists (and invertebrates), bacteria and fungi were characterized using the primer sets TAReuk454FWD1/TAReukREV3 to amplify 18S rRNA gene (Stoeck et al., 2010), 515F/806R for 16S rRNA gene (Bates et al., 2011), and ITS1F/ITS2R for ITS region (Kawasaki, 1990), respectively. The amplicons were purified, quantified, and sequenced on the Illumina MiSeq PE 300 platform (Illumina). Paired‐end reads were merged and filtered to remove those with a low Phred Quality Score (*Q* < 20), short length (<100 nt), and barcodes. Chimeric sequences were removed by the USEARCH tool with the UCHIME algorithm (Edgar et al., 2011). We used Quantitative Insights Into Microbial Ecology pipeline to analyse the generated high‐quality sequences (Caporaso et al., 2010).

Operational taxonomic units were clustered at 97% similarity. Taxonomy of bacteria, fungi, and protists plus invertebrates was assigned by using the SILVA database (V_138) (Quast et al., 2012), UNITE database 8.0 (Abarenkov et al., 2010), and Protist Ribosomal Reference (PR^2^) database (Guillou et al., 2012), respectively. The functional groups of protists (consumers, parasites, phototrophs, and unknown) were classified based on the feeding habits of protists at the genus level (Nguyen et al., 2020b, 2022). The protist taxa at the genus level are considered to have similar feeding modes. The nutrients of consumers are bacteria and other eukaryotes while phototrophs synthesize energies via photosynthesis. Parasites are protists living in soil animals or plants, and other unassigned protists are defined as unknown. Soil invertebrates (Metazoa) include Nematoda, Arthropoda, Rotifera, Tardigrada, Gastrotricha, Porifera, Annelida, and Platyhelminthes. In this study, we focused on the trophic effects of five most abundant classes of soil invertebrates (Nematoda, Arthropoda, Rotifera, Tardigrada, and Platyhelminthes), which are considered to have intimate interactions with protists in soil (Geisen et al., 2018; Potapov et al., 2022).

The trophic groups (bacterivores, fungivores, omnivores, and predators) of nematodes, excluding unassigned nematodes, herbivores (plant feeders), and animal parasites, were assigned at the genus level. In contrast to well‐documented aquatic eukaryotes, most of food and feeding habits (i.e., trophic guilds or groups) of other soil fauna have not been determined in the soil food web (Potapov et al., 2022). Hence, other soil invertebrates were classified into the genus level and family level for further analyses. There were a total of 4,198,680 protist sequences (19,438 sequences on average), 11,297,649 bacterial sequences (52,304 sequences on average), 4,622,957 fungal sequences (21,402 sequences on average), and 2,012,003 invertebrate sequences (9,315 sequences on average). To ensure evenness of sequencing depth across all samples, we rarefied the number of reads to 2,000 per sample for protists (18S rRNA gene), 25,000 for bacteria (16S rRNA gene), 32,000 for fungi (ITS region), and 210 for soil invertebrates (18S rRNA gene).

### Statistical analyses

Statistical analyses were conducted in the R platform and visualized in the “ggplot2” package (Wickham, 2016). The effects of biotic and abiotic factors (including soil properties, climatic factors, vegetation types, bacteria, fungi and invertebrates) on the alpha diversity (Shannon index) and community composition of soil protists were assessed by multiple regression and Mantel test (based on the Euclidean distances for abiotic factors and the Bray–Curtis dissimilarity matrices for soil organismic communities), respectively. The relationships of alpha diversity (Shannon index) of protists and their functional groups with that of other soil organisms (bacteria, fungi, and invertebrates) were estimated by the best polynomial fit model on the basis of the lowest corrected Akaike information criterion. To determine the effects of top‐down (i.e., invertebrates) and bottom‐up (i.e., bacteria and fungi) factors on the protist community and functional groups, Mantel tests were performed based on the Bray–Curtis community dissimilarity matrices.

For the co‐occurrence network, strong and significant correlations among microbial taxa at the genus level and invertebrates at the genus level were determined with Spearman correlation coefficients *ρ* ≥ 0.7 (positive) or *ρ* ≤ −0.7 (negative) and *p* < 0.001. The co‐occurrence network of protist and bacterial, fungal, and invertebrate taxa (at the genus level) was visualized in Cytoscape (https://cytoscape.org/). The names of organismic taxa were shown at the class level in the network. We further identified the biotic drivers for the relative abundances of dominant phyla and functional groups of soil protists by random forest modelling using the “randomForest” package (v. 4.6‐14) (Liaw & Wiener, 2002) and the “A3” package (Fortmann‐Roe, 2015). Effects of top‐down and bottom‐up factors were estimated by multiple regression based on the Bray–Curtis distance matrices in the “ecodist” package (Goslee & Urban, 2007).

## RESULTS

### Diversity and composition of soil protists and other organisms

**FIGURE 1:**
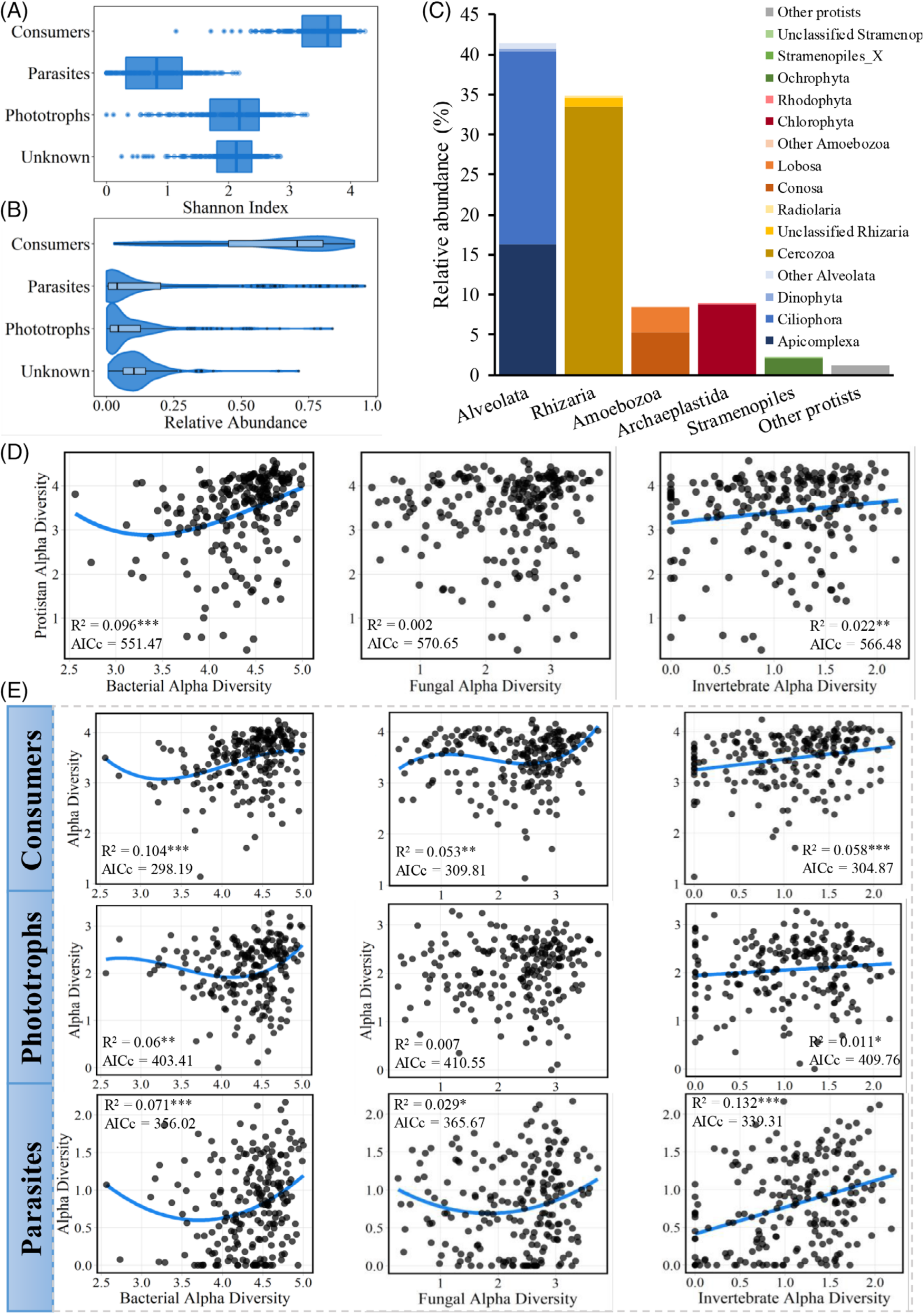
General diversity and composition of soil protist community. (A) Boxplots showing the alpha diversity (Shannon index) of protistan functional groups. (B) Violin plots and boxplots showing the relative abundances of protist functional groups. (C) Taxonomic composition of soil protists at the supergroup and phylum levels. The relationships between the alpha diversity of (D) protists and (E) their functional groups with top‐down (i.e., the alpha diversity of soil invertebrates) and bottom‐up factors (i.e., the alpha diversity of bacterial and fungal communities). *R* ^2^ indicates the percentage of variation in the alpha diversity estimated by the best polynomial fit model on the basis of the corrected Akaike information criterion (AICc). Significant relationships are indicated by: *p < 0.05; **p < 0.01; and ***p <0.001.

Consumers were the most diverse functional group of soil protists, followed by phototrophs and parasites (Figure 1A). Likewise, consumers were the most abundant group in the protist community (61.47% of total abundance), followed by parasites (17.00%) and phototrophs (9.88%) (Figure 1B). Alveolata (41.24%), Rhizaria (34.65%), and Archaeplastida (8.91%) were the most abundant supergroups (Figure 1C), which dominated parasitic, consumer and phototrophic groups of protists, respectively (Figure S2A). At the phylum level, Cercozoa (Rhizaria) and Ciliophora (Alveolata) constituted the majority of protists (Figure 1C) and consumers (Figure S2B). Meanwhile, parasites and phototrophs were comprised of the phyla Apicomplexa (15.62%) and Chlorophyta (8.83%), respectively (Figure S2B). For other trophic groups, bacteria were dominated by Actinobacteria, Proteobacteria, and Acidobacteria; fungi were dominated by Ascomycota and Basidiomycota; while soil invertebrates were dominated by Nematoda, Rotifera, and Arthropoda (Figure S3).

### Trophic drivers for the alpha diversity of soil protists

We quantified the influences of biotic and abiotic factors on the alpha diversity of the protist community. The regression analysis indicated that biotic factors, bacteria and invertebrates, had stronger effects on the protist diversity than climatic factors and soil properties (Figure S4A). The relationships between the alpha diversity of protists and their functional groups with top‐down (i.e., the alpha diversity of soil invertebrates) and bottom‐up factors (i.e., the alpha diversity of bacteria and fungi) were further estimated by the best polynomial fit model. We found that the alpha diversity of the whole protist community was best associated with the bacterial diversity (*R* ^2^ = 0.096, *p* < 0.001), but to a lesser extent with that of invertebrates (*R* ^2^ = 0.022, *p* < 0.01) (Figure 1D). The diversity of consumers and phototrophs had stronger relationships with the bacterial diversity (*R* ^2^ = 0.104 and *R* ^2^ = 0.060, respectively) than with the invertebrate diversity (*R* ^2^ = 0.058 and *R* ^2^ = 0.011, respectively), while the parasitic diversity was more affected by soil invertebrates (*R* ^2^ = 0.132) than by bacteria (*R* ^2^ = 0.071) (Figure 1E). The fungal community had no significant effects on the diversity of the whole protist community or phototrophs (*p* > 0.05).

### Trophic interrelationships between protists and other soil organisms

**FIGURE 2:**
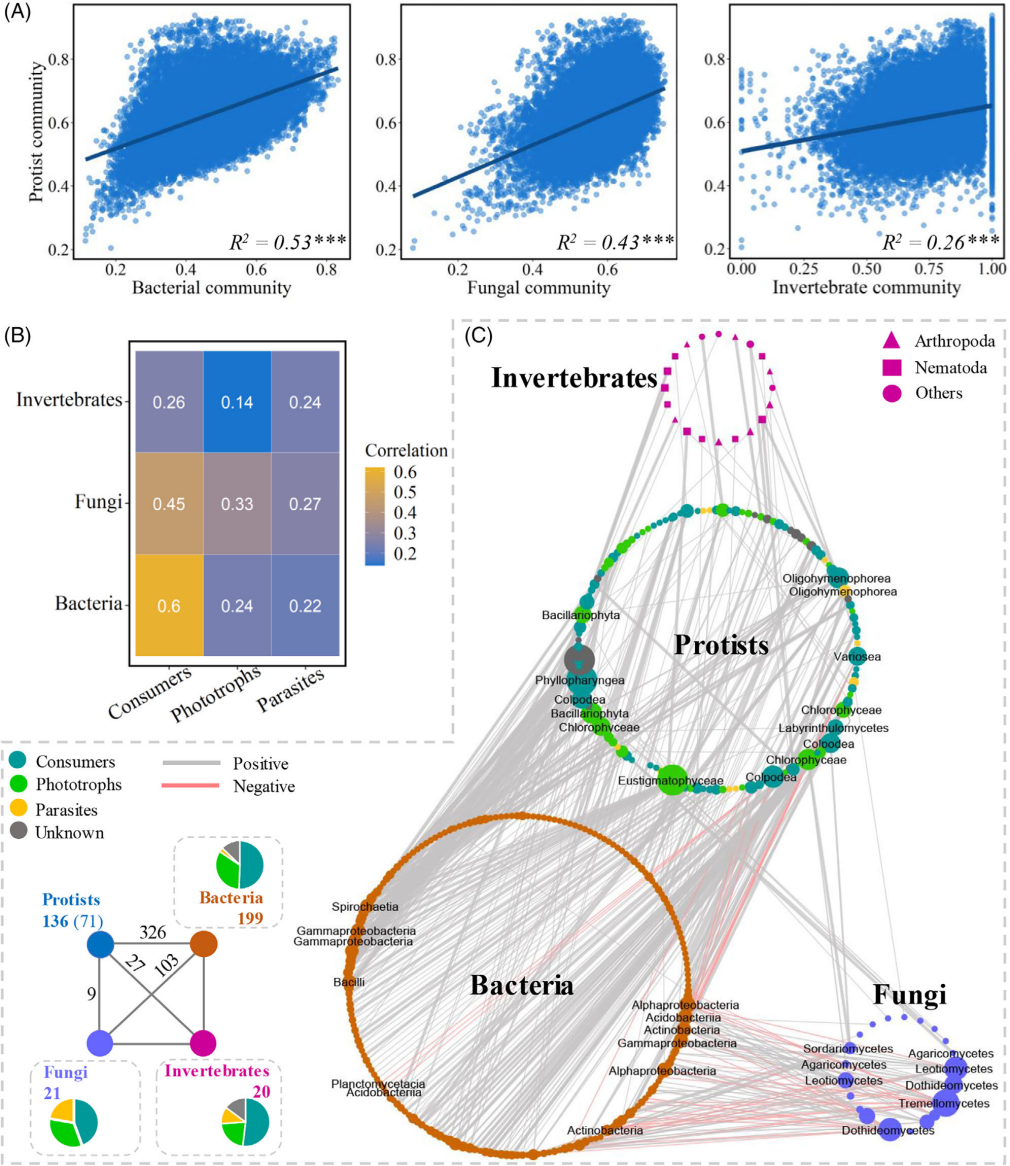
Protist community composition had strong associations with bottom‐up factors. The relationships of top‐down and bottom‐up factors with the compositions of (A) the protist community and (B) their main functional groups determined by Mantel tests based on the Bray–Curtis dissimilarity. Significant associations are indicated by: *p < 0.05; **p < 0.01; and ***p <0.001. (C) Co‐occurrence network of protists and other soil organisms within soil food webs. Only cross‐group interactions between organismic taxa are shown. A connection indicates a strong and significant Spearman correlation, divided into positive (*ρ* ≥ 0.7, *p* < 0.001; grey) or negative (*ρ* ≤ −0.7, *p* < 0.001; red) edges. Each node represents a taxon at the genus level and is displayed by its class level. The size of node is proportional to the number of connections (edge count). The thickness of edges is proportional to Spearman correlation coefficients (i.e., the thicker edges denote the stronger trophic interrelationships). A summary of node–edge statistics is provided at the bottom left of the network. Pie charts represent the percentage of interactions between functional groups and other soil organisms. The number in backets denotes the number of inner connections among protistan functional taxa.

We also assessed the importance of numerous biotic and abiotic factors for the community structure of soil protists. Mantel test revealed the protist communities across natural soil ecosystems were best explained by biotic factors bacterial (*R* ^2^ = 0.53, *p* < 0.001), fungal (*R* ^2^ = 0.43), and invertebrate communities (*R* ^2^ = 0.26) (Figures 2A and S4B). Similar to the whole protist community, consumers were more associated with their microbial prey bacteria (*R* ^2^ = 0.60, *p* < 0.001) and fungi (*R* ^2^ = 0.45), to a lesser extent with invertebrates (*R* ^2^ = 0.26). Meanwhile, both phototrophic and parasitic groups were best predicted by fungi (*R* ^2^ = 0.33 and *R* ^2^ = 0.27, respectively; *p* < 0.001), followed by the effects of bacteria on phototrophs (*R* ^2^ = 0.24) and invertebrates on parasites (*R* ^2^ = 0.24) (Figure 2B). Notably, soil invertebrates were slightly correlated with the phototrophic community (*R* ^2^ = 0.14, *p* < 0.001).

Through the co‐occurrence network analysis, we further disentangled the interrelationships between protists and other soil organismic taxa at the genus level. There were a total of 536 robust connections among 376 microbial taxa (Figure 2C). We observed 326 cross‐group connections between protistan and bacterial taxa, 27 protist‐invertebrate connections and 9 protist‐fungi connections. In functional groups of protists, consumers had the highest number of trophic interactions with other organisms (Figure 2C), accounting for 48.97% of cross‐interactions of protists (Table S2), followed by phototrophs. Most of protistan lineages were positively interrelated with other lineages, especially protist‐fungi and protist‐invertebrate interactions (mostly with Nematoda and Arthropoda). However, there were 14 strong and negative associations detected between protists and bacteria, including consumer taxa Variosea (Conosa) and Colpodea (Ciliophora) with the dominant bacterial lineages Actinobacteria (Actinobacteria), Alphaproteobacteria and Gammaproteobacteria (Proteobacteria), Acidobacteria (Acidobacteria), and Planctomycetacia (Planctomycetes) (Figure 2C).

### Trophic drivers for the community structure of protists

**FIGURE 3:**
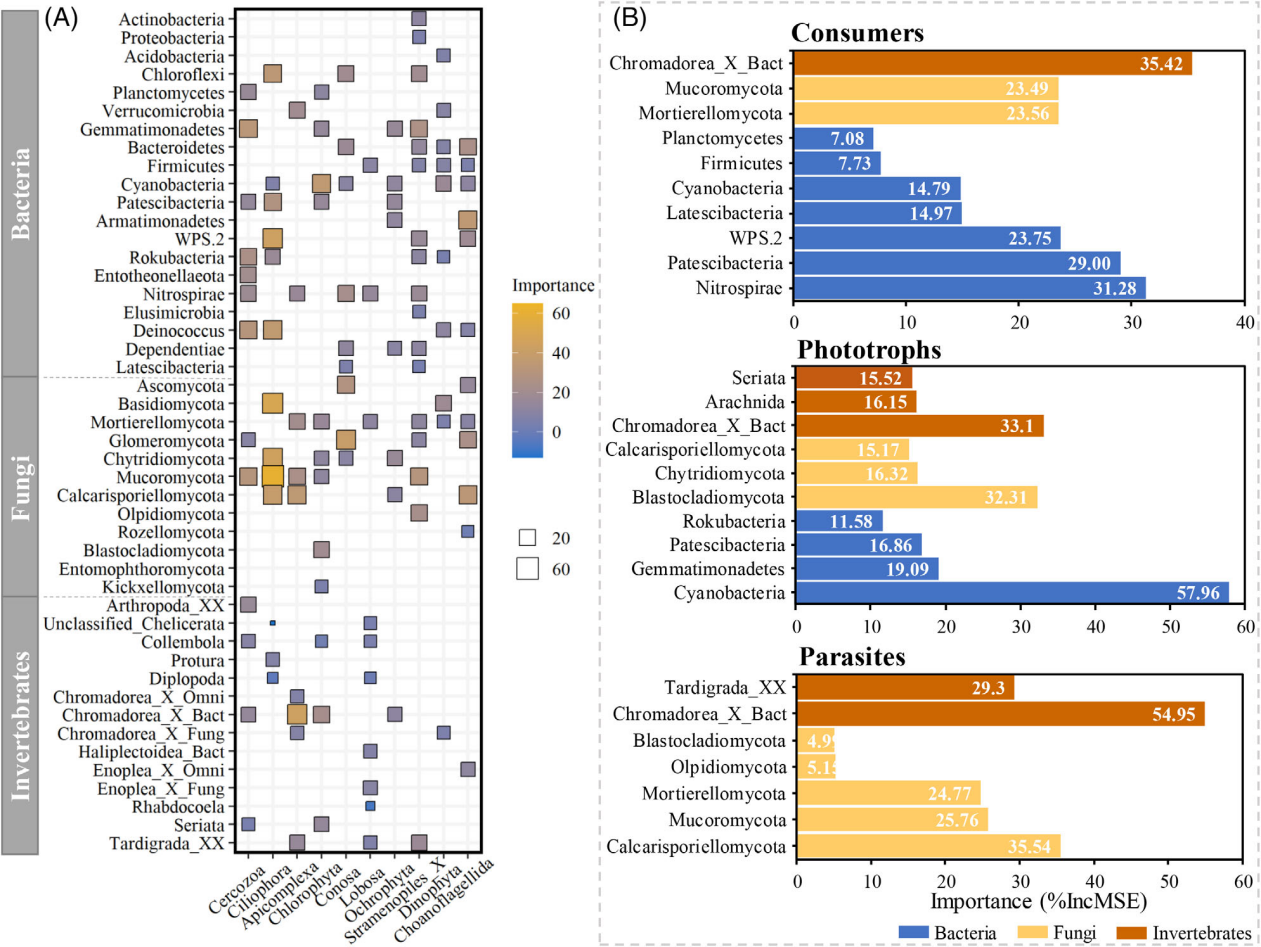
Importance of microbial and invertebrate taxa in predicting the abundances of (A) taxonomic (at the phylum level) and (B) functional groups of soil protists. Squares in a heatmap and bar charts represent the importance (i.e., %IncMSE) of significant biotic predictors (*p* < 0.05) in explaining the variation in protist abundance as revealed by random forest modelling. Uncorrelated taxa are not shown.

Random forest modelling further indicated that many bacterial and fungal phyla were the most important predictors of protistan phyla (Figure 3A). Dominant phyla of protists, Cercozoa, Cilliophora, and Conosa, which mostly function as consumers, were strongly driven by microbial prey bacteria and fungi. The abundance of Cercozoa was best predicted by bacterial taxa Gemmatimonadetes and Deinococcus, and a fungal taxon Mucoromycota. Fungi (e.g., Mucoromycota, Basidiomycota and Chytridiomycota) had the most important influence on Ciliophora, followed by bacterial phyla. Moreover, many invertebrate (bacterivorous Chromadorea_X) and fungal phyla were also top predictors of Apicomplexa and Stramenopiles_X—as parasitic representatives of protists. Although invertebrates played a less important role in predicting protists' taxonomic abundance than microbial taxa, many taxa of Arthropoda (e.g., Arthropoda_XX and Collembola), Nematoda (Chromadorea_X and Enoplea_X), Platyhelminthes (Seriata), and Tardigrada significantly affected several dominant phyla of protists. Phototrophic phyla were also driven by invertebrates Chromadorea_X, Seriata and Collembola.

Furthermore, random forest modelling revealed that consumers were best predicted by a bacterivorous nematode (Chromadorea_X) and many bacterial phyla (Nitrospirae, Patescibacteria, and WPS.2), followed by fungal lineages (Figure 3B). Many taxa of bottom‐up factors contributed to controlling the protist consumer community. Meanwhile, Cyanobacteria (bacteria), Chromadorea_X (invertebrate), and Blastocladiomycota (fungi) were the top three predictors of phototrophic protists. Invertebrates (Chromadorea_X and Tardigrada_XX) and fungi (e.g., Calcarisporiellomycota, Mucoromycota, and Mortierellomycota) were the primary drivers of protist parasites. In particular, fungi and invertebrates dominantly regulated the parasitic group. No significant effects of bacterial phyla were observed on the parasitic abundance.

**FIGURE 4:**
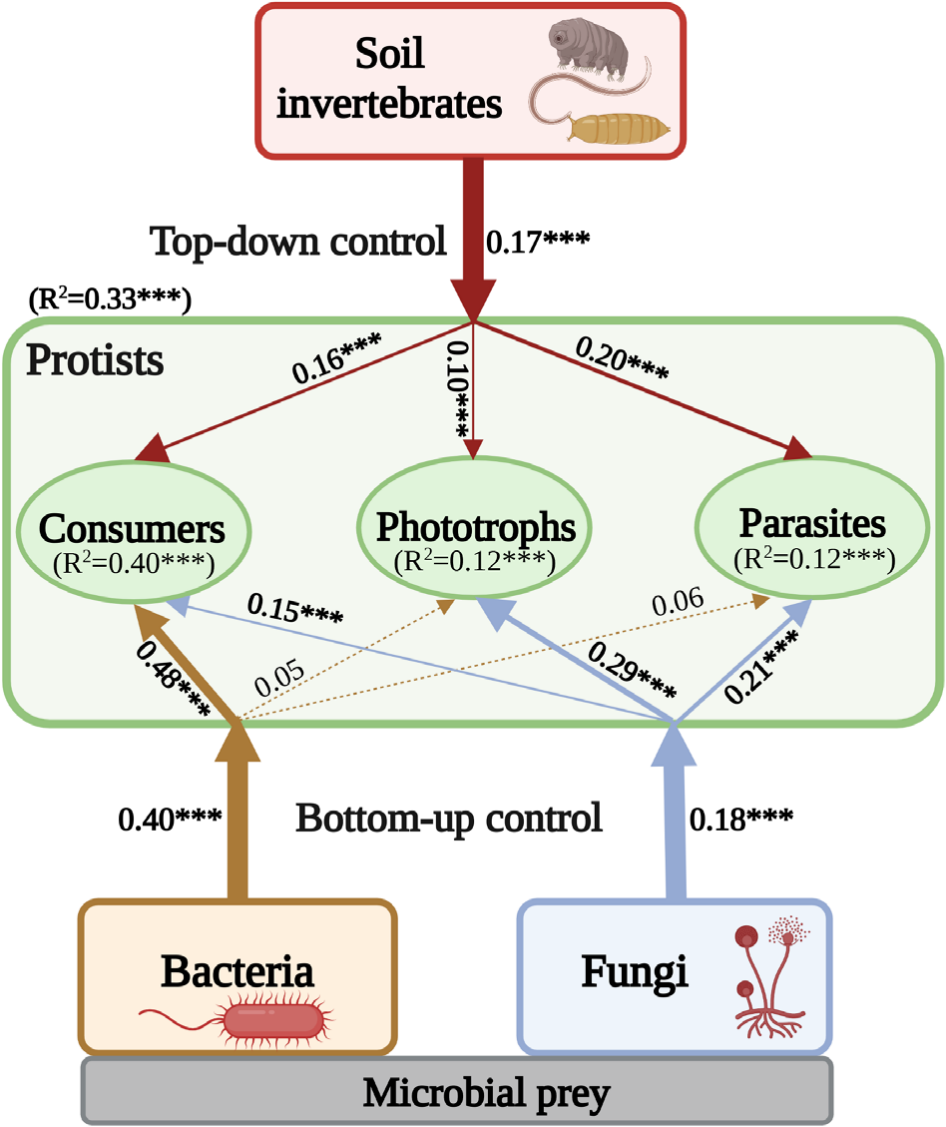
A diagram illustrates the trophic regulations on soil protist community and their functional groups. Effects of top‐down and bottom‐up factors were estimated by multiple regression based on the Bray–Curtis distance matrices. Solid and dashed arrows indicate significant and nonsignificant relationships, respectively. Numbers adjacent to the arrows are path coefficients of the relationship between protistan functional groups and other organisms, and *R* ^2^ indicates the variance explained. Significant relationships are indicated by: *p < 0.05; **p < 0.01; and ***p < 0.001.

Finally, we estimated the overall effects of trophic regulations on the community composition of protists and their functional groups. The multiple regression analysis indicated that the protist community was best shaped by bacteria, and to a weaker extent, by fungi and invertebrates (overall explanatory variation: *R* ^2^ = 0.33, *p* < 0.001; Figure 4). Likewise, a similar pattern was observed for the community structure of consumers, with bacteria being the most important factor, followed by fungi and invertebrates (*R* ^2^ = 0.40, *p* < 0.001). Notably, fungal community was the primary factor driving the phototrophic composition, followed by top‐down effects of soil invertebrates (*R* ^2^ = 0.12, *p* < 0.001). For parasitic protists, their community composition was best explained by fungal and invertebrate communities (*R* ^2^ = 0.12, *p* < 0.001).

## DISCUSSION

Soil protists, occupying different trophic positions, are important microbial regulators and consumers of bacteria and fungi in the soil food web. In the energy channel, protists serve as nutrient resources for organisms at higher trophic levels (e.g., soil invertebrates). The top‐down control by high trophic levels and bottom‐up control by lower trophic levels, can influence the population of soil organisms (Leroux & Loreau, 2015). The impacts of climatic and edaphic properties, biological factors, and vegetation types on soil protists have been reported in regional and global scale studies (Bates et al., 2013; Chen et al., 2022, 2021; Nguyen et al., 2021; Oliverio et al., 2020). However, no studies have attempted to compare the relative contributions of bottom‐up and top‐down regulators to the distribution and structure of the soil protist community. Our study addresses this key knowledge gap by disentangling the relative importance of bottom‐up and top‐down regulations in shaping the diversity and compositions of soil protists and their functional groups in natural soil ecosystems.

We identified the great role of bottom‐up control by bacteria in driving both the alpha diversity and composition of soil protists in natural habitats, which is in line with our hypothesis. Bacteria are renowned as the primary and favourite nutrients of protistan consumers, including bacterivores (feeding on bacteria) and omnivores (feeding on bacteria and eukaryotes) (Geisen et al., 2018). The strong bottom‐up effect of bacteria on protists can be derived from (i) the predominance of consumers across all soil samples (61.47% of total abundance) (Figures 1B and S2) and the feeding preference of protistan consumers on bacteria; (ii) the symbiosis of many consumers with bacteria in performing soil functions and environmental adaptation (Gast et al., 2009); and (iii) shared water‐filled space within the soil matrix. Although fungi and arthropods occupy air‐filled pores, protists and bacteria actively inhabit similar‐size soil pores and water‐filled soil microsites (Nielsen, 2019). Hence, the frequent encounter between protists and bacteria may be one of the reasons which render the effects of bacteria on soil protists. In addition, top‐down effects of invertebrates, particularly the nematode Chromadorea_X, also strongly shaped soil protists through (i) the direct predation of protists by nematodes, rotifera, tardigrades and arthropods; and (ii) indirect cascade effects of invertebrates on protists by feeding or changing microbial prey (Potapov et al., 2022).

The bottom‐up role of bacteria is stronger in the consumer community since bacterivores feeding on bacteria are considered as the most dominant and prevalent protist consumers in soils (Geisen, 2016). Moreover, many consumers and bacteria form symbiotic relationships in element cycling (e.g., with Cyanobacteria in nitrogen fixation) and/or environmental adaptation (e.g., anoxic or oligotrophic conditions) (Gast et al., 2009). The highest number of robust connections was found between protists and bacterial taxa among all estimated trophic levels (Figure 2B,C), and many bacterial phyla were identified as strong predictors of the consumer group (Figure 3B). These findings further support the strong effect of bacteria on protistan consumers. The intensive protist‐bacteria associations were also recorded across different biomes (Oliverio et al., 2020) and agricultural ecosystems (Chen et al., 2021), but effects of other trophic levels were not covered in these studies. Interestingly, we identified that the relative abundance of consumers was best predicted by the bacterivorous nematode Chromadorea_X, which can be explained by (i) indirect cascade effects of Chromadorea_X on protists by feeding or changing bacterial population (Potapov et al., 2022); and (ii) the shared habitat preference between bacterivorous nematodes and protists due to their feeding preference on bacteria. Therefore, our results filled the knowledge gap in effects of multiple trophic levels in previous studies.

Although the importance of many invertebrates on phototrophic protists was identified, the phototrophic diversity and structure were most affected by bacteria and fungi, respectively. Strikingly, Cyanobacteria, rather than invertebrate taxa, was the best predictor of Chlorophyta and phototrophic community (Figure 3). The strong effects of microbial prey on phototrophs may derive from (i) their symbiotic relationships in performing soil multifunction and (ii) their shared active space in soil. Phototrophs function as carbon fixers and form symbiotic partnerships with bacteria, especially Cyanobacteria, in biogeochemical cycles and various functions in terrestrial and aquatic systems (Ramanan et al., 2016; Subashchandrabose et al., 2013). Recent studies also reported the reciprocal symbiosis between phototrophs and fungi (Picard et al., 2013), for example, the interaction between an algae *Nannochloropsis oceanica* and a soil fungus *Mortierella elongate* in nutrient exchange and oil production (Du et al., 2019). Furthermore, we identified the importance of invertebrates in regulating the parasitic diversity (Figure 1E) and the strong influence of fungi on their community composition (Figure 4). Nematoda and Tardigrada significantly predicted parasitic protists (Figure 3A,B). Many protists are renowned as parasites of plants, animals, and fungi (Geisen et al., 2018). The strong associations of invertebrates with parasites can be explained by the mutual influences: parasites can infect invertebrates as their hosts via top‐down control, in turn they are regulated by bottom‐up effects of the hosts due to their nutrient and habitat dependance. Besides, fungi were the best drivers of the parasitic community composition, interpreted by the parasitism of some protists on fungi (Geisen et al., 2018).

Our findings demonstrate the crucial role of biotic factors in shaping the communities of soil protists, which implies the importance of trophic regulations in interpreting soil functions and ecosystem services in future studies. The strong interrelationships between protists and other organisms in our work indicate the great contribution of trophic interactions to shaping the community structure of soil protists and other organisms at large scales, such as the importance of protists in shaping bacterial (Asiloglu et al., 2021) and fungal communities (Huang et al., 2021). In particular, the highest number of protist‐bacteria connections within soil microbiome will facilitate the interpretation of their roles in the evolution of antimicrobial resistance (Nguyen et al., 2020a), litter decomposition and carbon cycling (Geisen et al., 2021), and other essential functions. Nevertheless, there are some limitations in our study: first, we did not assess the effects of trophic cascades from microbes‐invertebrate interactions on soil protists and their functional traits; second, because nematodes are also one of energy sources of protists, bottom‐up control of soil nematodes is needed to disentangle in future research; finally, the use of universal primer sets targeting 18S rRNA genes in our study is not optimal to characterize invertebrates and protists, and thus there is a call for the development of new primers and further in‐depth study of their identity and functional traits.

## AUTHOR CONTRIBUTIONS

**Thi Bao Anh Nguyen:** Data curation (equal); formal analysis (equal); investigation (equal); methodology (equal); visualization (equal); writing – original draft (equal); writing – review and editing (equal). **Qing‐Lin Chen:** Writing – review and editing (equal). **Zhen‐Zhen Yan:** Writing – review and editing (equal). **Chaoyu Li:** Writing – review and editing (equal). **Ji‐Zheng He:** Supervision (equal); writing – review and editing (equal). **Hang‐Wei Hu:** Conceptualization (equal); funding acquisition (equal); writing – original draft (equal); writing – review and editing (equal).

## CONFLICT OF INTEREST

The authors declare no conflict of interest.

## Supporting information


**FIGURE S1.** The geographic locations of 72 sampling sites across eastern Australia.
**FIGURE S2.** Composition of functional groups at (A) supergroup and (B) phylum levels.
**FIGURE S3.** Relative abundance of dominant phyla of bacteria, fungi and invertebrates.
**FIGURE S4.** Effects of environmental factors on the alpha diversity (A) and community composition of soil protists estimated by multiple regression and Mantel test, respectively. Significant relationships are indicated by: *p < 0.05, **p < 0.01; and ***p < 0.001.
**TABLE S1.** List of 72 sampling sites in Australia.
**Table S2.** The percentage of cross‐group interactions between functional groups of protists and other soil organisms.

## Data Availability

All raw sequencing data of bacteria, fungi and eukaryotes were deposited at the National Centre for Biotechnology Information Sequence Read Archive under the accession number PRJNA891463, PRJNA891464, and PRJNA891465, respectively.
