# Supplementary material for: Trophic interrelationships of bacteria are important for shaping soil protist communities
Source: Environ Microbiol Rep. 2023 Mar 29;15(4):298–307. doi: 10.1111/1758-2229.13143 (PMC10316372; doi:10.1111/1758-2229.13143)
Supplement: Supplementary file 1 — FIGURE S1. The geographic locations of 72 sampling sites across eastern Australia. FIGURE S2. Composition of functional groups at (A) supergroup and (B) phylum levels. FIGURE S3. Relative abundance of dominant phyla of bacteria, fungi and invertebrates. FIGURE S4. Effects of environmental factors on the alpha diversity (A) and community composition of soil protists estimated by multiple regression and Mantel test, respectively. Significant relationships are indicated by: *p < 0.05, **p < 0.01; and ***p < 0.001. TABLE S1. List of 72 sampling sites in Australia. Table S2. The percentage of cross‐group interactions between functional groups of protists and other soil organisms. [file EMI4-15-298-s001.docx]

***Supporting Information***

*Title*

**Trophic interrelationships of bacteria are important for shaping soil protist communities**

Thi Bao Anh Nguyen^1^, Qing-Lin Chen^1^, Zhen-Zhen Yan^1^, Chaoyu Li^1^, Ji-Zheng He^1^, Hang-Wei Hu^1,^*

^1^ School of Agriculture and Food, Faculty of Science, the University of Melbourne, Parkville VIC 3010, Australia.

For correspondence:

Hang-Wei Hu,

Email: [hang-wei.hu@unimelb.edu.au](mailto:hang-wei.hu@unimelb.edu.au).

Address: School of Agriculture and Food, Faculty of Science, the University of Melbourne, Parkville VIC 3010, Australia.

# **Figure S1.** The geographic locations of 72 sampling sites across eastern Australia.


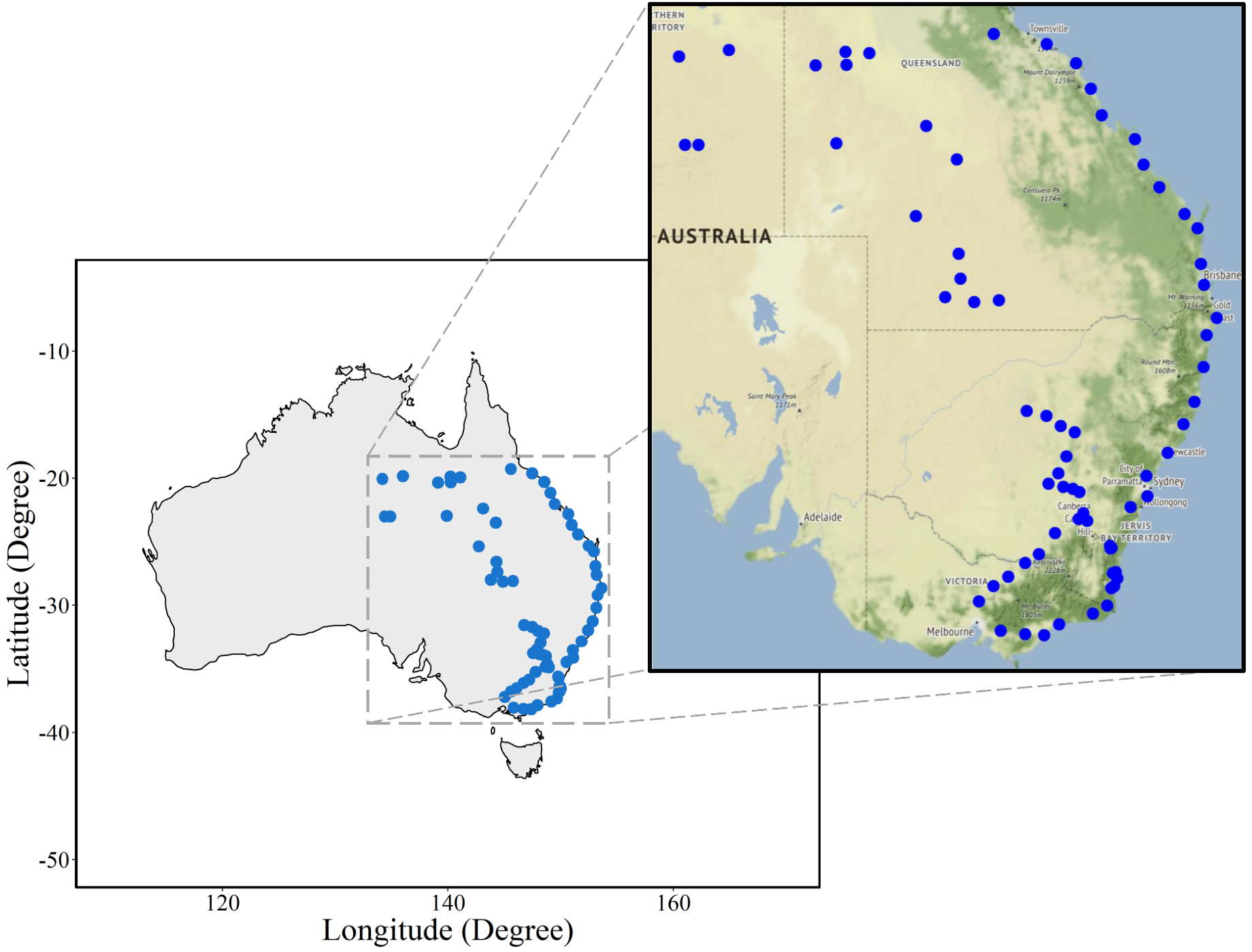


# **Figure S2.** Composition of functional groups at (A) supergroup and (B) phylum levels.


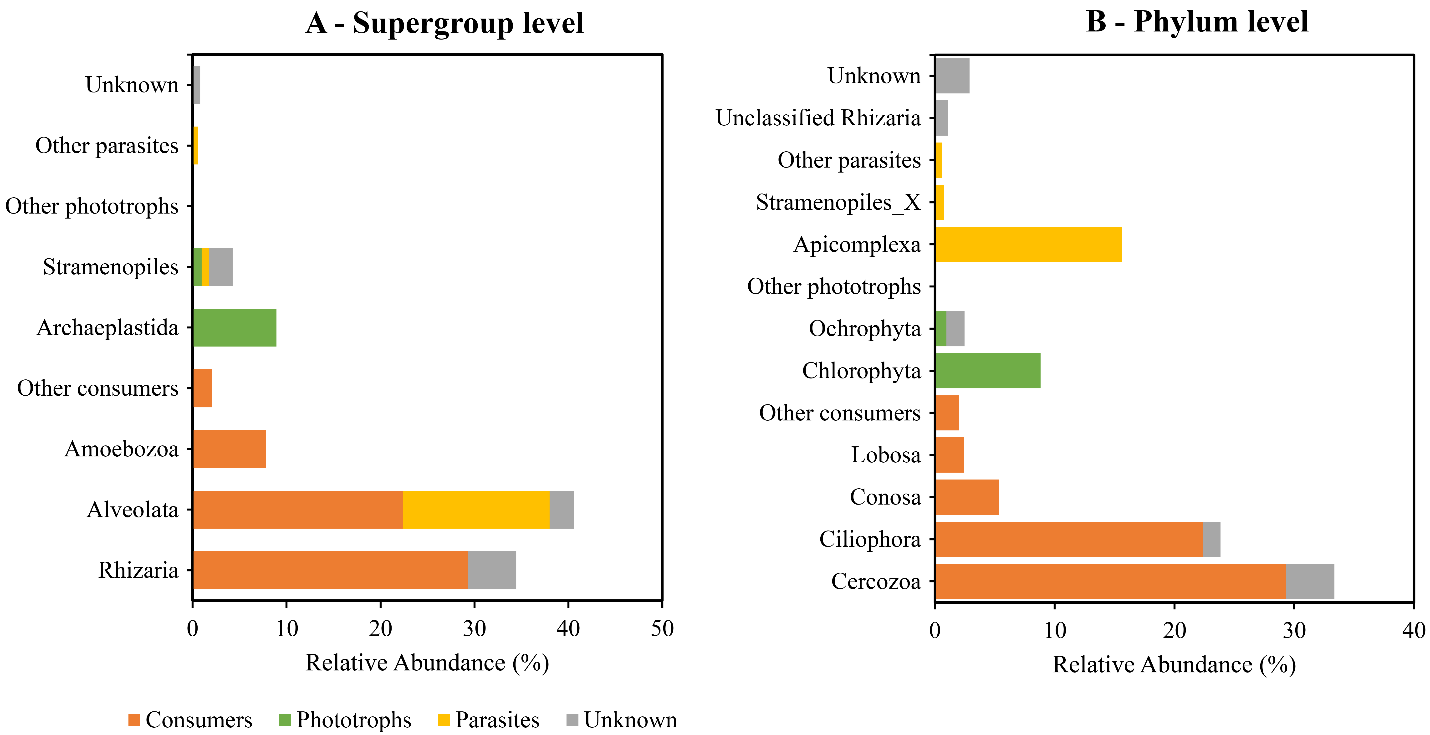


# **Figure S3.** Relative abundance of dominant phyla of bacteria, fungi and invertebrates.


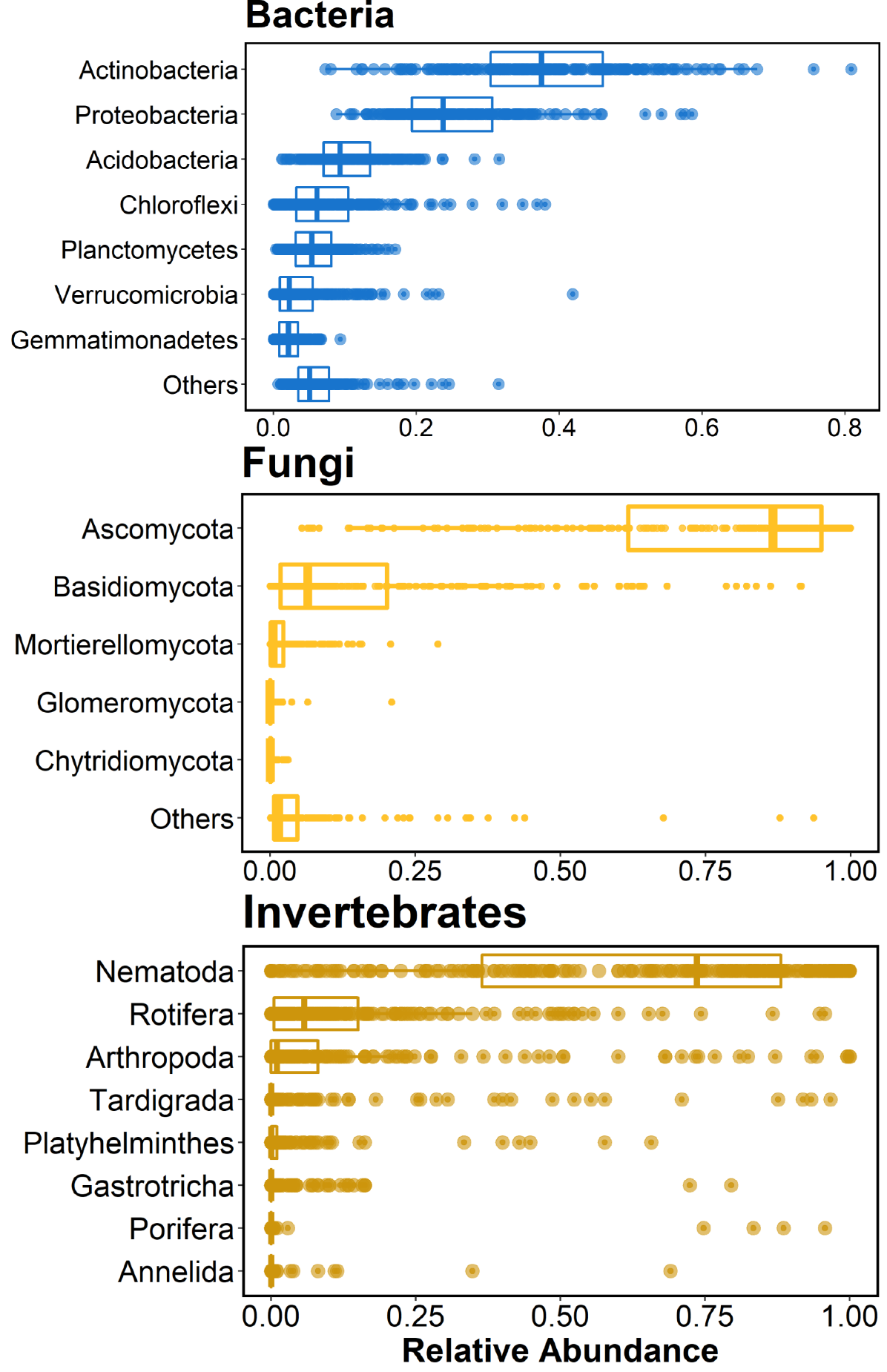


# **Figure S4.** Effects of environmental factors on the alpha diversity (A) and community composition of soil protists estimated by multiple regression and Mantel test, respectively. Significant relationships are indicated by: *P* values < 0.05*, < 0.01** and < 0.001***.


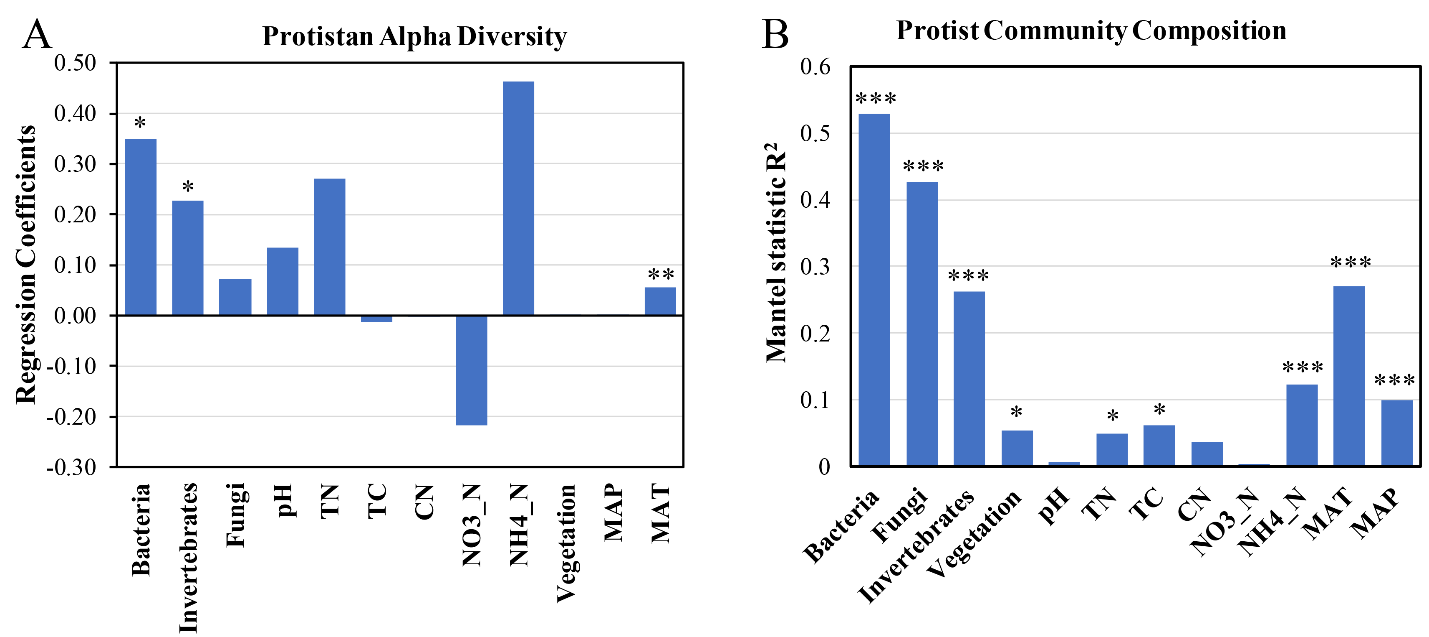


# **Table S1**. List of 72 sampling sites in Australia.

| **No.** | **Sample ID** | **Latitude** | **Longitude** | **Vegetation** | **Soil pH** | **TN (g/kg)** | **TC (g/kg)** | **C/N** | **NO_3_^-^-N (g/kg)** | **NH_4_^+^-N (g/kg)** | **MAP (mm)** | **MAT (℃)** |
| --- | --- | --- | --- | --- | --- | --- | --- | --- | --- | --- | --- | --- |
| 1 | S1 | -38.0561 | 145.8189 | Native forests and woodlands | 5.42 | 3.041 | 50.125 | 16.481 | 0.032 | 0.005 | 977 | 13.9 |
| 2 | S2 | -38.0561 | 145.8189 | Native forests and woodlands | 5.35 | 4.046 | 72.315 | 17.871 | 0.050 | 0.007 | 977 | 13.9 |
| 3 | S3 | -38.0561 | 145.8189 | Native forests and woodlands | 5.31 | 3.370 | 57.448 | 17.046 | 0.047 | 0.002 | 977 | 13.9 |
| 4 | S7 | -38.1505 | 146.7047 | Native forests and woodlands | 5.62 | 2.684 | 36.324 | 13.533 | 0.033 | 0.029 | 698 | 13.7 |
| 5 | S8 | -38.1505 | 146.7047 | Native forests and woodlands | 5.61 | 2.762 | 40.140 | 14.534 | 0.011 | 0.025 | 698 | 13.7 |
| 6 | S9 | -38.1505 | 146.7047 | Native forests and woodlands | 5.63 | 2.733 | 38.456 | 14.072 | 0.012 | 0.009 | 698 | 13.7 |
| 7 | S13 | -38.1858 | 147.3847 | Native forests and woodlands | 6.35 | 0.843 | 19.380 | 22.992 | 0.001 | 0.018 | 645 | 14 |
| 8 | S14 | -38.1858 | 147.3847 | Native forests and woodlands | 6.25 | 0.581 | 11.981 | 20.610 | 0.001 | 0.007 | 645 | 14 |
| 9 | S15 | -38.1858 | 147.3847 | Native forests and woodlands | 6.19 | 0.808 | 16.381 | 20.270 | 0.002 | 0.009 | 645 | 14 |
| 10 | S19 | -37.8718 | 147.9351 | Native forests and woodlands | 4.79 | 3.006 | 41.794 | 13.904 | 0.047 | 0.005 | 732 | 14.3 |
| 11 | S20 | -37.8718 | 147.9351 | Native forests and woodlands | 5.01 | 2.006 | 28.528 | 14.222 | 0.023 | 0.001 | 732 | 14.3 |
| 12 | S21 | -37.8718 | 147.9351 | Native forests and woodlands | 5.02 | 2.050 | 27.225 | 13.280 | 0.025 | 0.002 | 732 | 14.3 |
| 13 | S25 | -37.57 | 149.15 | Native grasslands | 5.28 | 2.782 | 60.386 | 21.708 | 0.027 | 0.011 | 1014 | 14.2 |
| 14 | S26 | -37.57 | 149.15 | Native grasslands | 5.11 | 4.282 | 90.116 | 21.045 | 0.031 | 0.015 | 1014 | 14.2 |
| 15 | S27 | -37.57 | 149.15 | Native grasslands | 5.58 | 2.648 | 56.414 | 21.307 | 0.013 | 0.037 | 1014 | 14.2 |
| 16 | S28 | -37.3333 | 149.6667 | Native forests and woodlands | 5.79 | 0.596 | 15.408 | 25.874 | 0.001 | 0.011 | 929 | 14.1 |
| 17 | S29 | -37.3333 | 149.6667 | Native forests and woodlands | 5.42 | 0.718 | 18.881 | 26.285 | 0.001 | 0.005 | 929 | 14.1 |
| 18 | S30 | -37.3333 | 149.6667 | Native forests and woodlands | 5.23 | 1.143 | 25.450 | 22.264 | 0.007 | 0.060 | 929 | 14.1 |
| 19 | S31 | -36.8388 | 149.8152 | Native shrublands | 5.01 | 4.364 | 67.246 | 15.411 | 0.030 | 0.003 | 961 | 13.7 |
| 20 | S32 | -36.8388 | 149.8152 | Native shrublands | 5.04 | 1.371 | 25.221 | 18.403 | 0.001 | 0.009 | 961 | 13.7 |
| 21 | S33 | -36.8388 | 149.8152 | Native shrublands | 4.73 | 1.147 | 24.602 | 21.449 | 0.001 | 0.010 | 961 | 13.7 |
| 22 | S34 | -36.77876 | 149.92166 | Native shrublands | 5.94 | 1.458 | 21.262 | 14.586 | 0.013 | 0.001 | 915 | 15 |
| 23 | S35 | -36.77876 | 149.92166 | Native shrublands | 6.15 | 1.239 | 17.722 | 14.304 | 0.009 | 0.001 | 915 | 15 |
| 24 | S36 | -36.77876 | 149.92166 | Native shrublands | 6.07 | 1.404 | 21.190 | 15.093 | 0.014 | 0.001 | 915 | 15 |
| 25 | S37 | -36.5484 | 150.0345 | Native forests and woodlands | 5.71 | 6.491 | 93.931 | 14.471 | 0.025 | 0.031 | 958 | 15.4 |
| 26 | S38 | -36.5484 | 150.0345 | Native forests and woodlands | 5.61 | 3.711 | 70.324 | 18.950 | 0.002 | 0.006 | 958 | 15.4 |
| 27 | S39 | -36.5484 | 150.0345 | Native forests and woodlands | 5.49 | 6.595 | 112.175 | 17.009 | 0.023 | 0.015 | 958 | 15.4 |
| 28 | S40 | -36.3968 | 149.8721 | Native forests and woodlands | 5.88 | 2.271 | 34.843 | 15.340 | 0.005 | 0.007 | 1005 | 15 |
| 29 | S41 | -36.3968 | 149.8721 | Native forests and woodlands | 5.70 | 1.963 | 30.624 | 15.604 | 0.005 | 0.013 | 1005 | 15 |
| 30 | S42 | -36.3968 | 149.8721 | Native forests and woodlands | 5.81 | 2.096 | 32.318 | 15.420 | 0.012 | 0.003 | 1005 | 15 |
| 31 | S43 | -36.3647 | 149.9729 | Native forests and woodlands | 5.67 | 2.634 | 38.208 | 14.508 | 0.015 | 0.001 | 1005 | 15 |
| 32 | S44 | -36.3647 | 149.9729 | Native forests and woodlands | 5.36 | 2.513 | 37.111 | 14.770 | 0.035 | 0.006 | 1005 | 15 |
| 33 | S45 | -36.3647 | 149.9729 | Native forests and woodlands | 5.50 | 2.651 | 55.314 | 20.867 | 0.022 | 0.002 | 1005 | 15 |
| 34 | S52 | -35.60298 | 149.76579 | Native forests and woodlands | 5.96 | 2.521 | 36.268 | 14.386 | 0.006 | 0.001 | 977 | 12.6 |
| 35 | S53 | -35.60298 | 149.76579 | Native forests and woodlands | 5.96 | 3.619 | 50.862 | 14.053 | 0.009 | 0.002 | 977 | 12.6 |
| 36 | S54 | -35.60298 | 149.76579 | Native forests and woodlands | 5.95 | 2.346 | 32.381 | 13.806 | 0.011 | 0.001 | 977 | 12.6 |
| 37 | S55 | -35.61254 | 149.77861 | Native forests and woodlands | 5.74 | 2.492 | 26.558 | 10.656 | 0.029 | 0.001 | 977 | 12.6 |
| 38 | S56 | -35.61254 | 149.77861 | Native forests and woodlands | 5.72 | 2.867 | 30.730 | 10.719 | 0.037 | 0.001 | 977 | 12.6 |
| 39 | S57 | -35.61254 | 149.77861 | Native forests and woodlands | 5.68 | 2.533 | 27.852 | 10.997 | 0.041 | 0.001 | 977 | 12.6 |
| 40 | S58 | -35.66259 | 149.82275 | Native grasslands | 6.00 | 0.560 | 6.641 | 11.869 | 0.009 | 0.001 | 977 | 12.6 |
| 41 | S59 | -35.66259 | 149.82275 | Native grasslands | 6.37 | 0.696 | 8.725 | 12.538 | 0.011 | 0.001 | 977 | 12.6 |
| 42 | S60 | -35.66259 | 149.82275 | Native grasslands | 6.16 | 1.353 | 18.200 | 13.451 | 0.038 | 0.001 | 977 | 12.6 |
| 43 | S61 | -35.67615 | 149.78953 | Native grasslands | 6.03 | 2.586 | 38.764 | 14.993 | 0.056 | 0.001 | 954 | 13.3 |
| 44 | S62 | -35.67615 | 149.78953 | Native grasslands | 6.21 | 3.229 | 44.366 | 13.739 | 0.057 | 0.001 | 954 | 13.3 |
| 45 | S63 | -35.67615 | 149.78953 | Native grasslands | 6.40 | 2.100 | 31.642 | 15.070 | 0.034 | 0.005 | 954 | 13.3 |
| 46 | S67 | -34.86359 | 148.94792 | Native grasslands | 5.40 | 5.367 | 77.210 | 14.385 | 0.148 | 0.034 | 746 | 13.3 |
| 47 | S68 | -34.86359 | 148.94792 | Native grasslands | 5.76 | 4.185 | 57.078 | 13.639 | 0.115 | 0.010 | 746 | 13.3 |
| 48 | S69 | -34.86359 | 148.94792 | Native grasslands | 5.92 | 3.889 | 53.925 | 13.868 | 0.082 | 0.011 | 746 | 13.3 |
| 49 | S70 | -34.64132 | 148.80715 | Native forests and woodlands | 5.03 | 2.763 | 41.810 | 15.132 | 0.032 | 0.002 | 737 | 13.3 |
| 50 | S71 | -34.64132 | 148.80715 | Native forests and woodlands | 5.03 | 2.115 | 34.180 | 16.164 | 0.031 | 0.017 | 737 | 13.3 |
| 51 | S72 | -34.64132 | 148.80715 | Native forests and woodlands | 5.09 | 2.142 | 35.948 | 16.780 | 0.023 | 0.010 | 737 | 13.3 |
| 52 | S76 | -34.00425 | 148.6645 | Native forests and woodlands | 5.85 | 1.697 | 23.518 | 13.856 | 0.020 | 0.001 | 718 | 14.3 |
| 53 | S77 | -34.00425 | 148.6645 | Native forests and woodlands | 5.81 | 1.972 | 27.107 | 13.749 | 0.018 | 0.001 | 718 | 14.3 |
| 54 | S78 | -34.00425 | 148.6645 | Native forests and woodlands | 5.79 | 2.206 | 33.069 | 14.991 | 0.015 | 0.001 | 718 | 14.3 |
| 55 | S79 | -33.9164 | 148.4259 | Native forests and woodlands | 6.22 | 2.227 | 35.907 | 16.127 | 0.011 | 0.001 | 747 | 14.6 |
| 56 | S80 | -33.9164 | 148.4259 | Native forests and woodlands | 6.28 | 1.910 | 28.938 | 15.150 | 0.016 | 0.001 | 747 | 14.6 |
| 57 | S81 | -33.9164 | 148.4259 | Native forests and woodlands | 5.73 | 2.901 | 42.192 | 14.546 | 0.176 | 0.022 | 747 | 14.6 |
| 58 | S82 | -33.8484 | 148.0845 | Native shrublands | 6.57 | 1.265 | 17.606 | 13.913 | 0.009 | 0.001 | 659 | 15.6 |
| 59 | S83 | -33.8484 | 148.0845 | Native shrublands | 6.57 | 1.323 | 16.888 | 12.762 | 0.009 | 0.001 | 659 | 15.6 |
| 60 | S84 | -33.8484 | 148.0845 | Native shrublands | 6.38 | 1.679 | 23.065 | 13.742 | 0.007 | 0.002 | 659 | 15.6 |
| 61 | S85 | -33.7585 | 147.5498 | Native forests and woodlands | 5.94 | 1.458 | 15.777 | 10.822 | 0.038 | 0.013 | 518 | 16.8 |
| 62 | S86 | -33.7585 | 147.5498 | Native forests and woodlands | 6.01 | 1.497 | 16.310 | 10.892 | 0.028 | 0.001 | 518 | 16.8 |
| 63 | S87 | -33.7585 | 147.5498 | Native forests and woodlands | 5.87 | 2.164 | 24.520 | 11.330 | 0.048 | 0.003 | 518 | 16.8 |
| 64 | S88 | -33.447 | 147.9059 | Native forests and woodlands | 6.41 | 1.321 | 16.448 | 12.454 | 0.004 | 0.009 | 568 | 16.9 |
| 65 | S89 | -33.447 | 147.9059 | Native forests and woodlands | 6.40 | 1.234 | 14.986 | 12.145 | 0.003 | 0.010 | 568 | 16.9 |
| 66 | S90 | -33.447 | 147.9059 | Native forests and woodlands | 6.26 | 2.139 | 24.796 | 11.591 | 0.011 | 0.007 | 568 | 16.9 |
| 67 | S91 | -32.93355 | 148.19605 | Native forests and woodlands | 6.18 | 3.150 | 34.102 | 10.826 | 0.028 | 0.001 | 603 | 16.8 |
| 68 | S92 | -32.93355 | 148.19605 | Native forests and woodlands | 6.43 | 3.037 | 33.534 | 11.042 | 0.037 | 0.001 | 603 | 16.8 |
| 69 | S93 | -32.93355 | 148.19605 | Native forests and woodlands | 6.10 | 2.670 | 29.855 | 11.184 | 0.026 | 0.001 | 603 | 16.8 |
| 70 | S94 | -32.1967 | 148.4975 | Native forests and woodlands | 6.18 | 2.801 | 46.194 | 16.493 | 0.017 | 0.001 | 626 | 17.3 |
| 71 | S95 | -32.1967 | 148.4975 | Native forests and woodlands | 6.40 | 3.475 | 58.387 | 16.802 | 0.025 | 0.001 | 626 | 17.3 |
| 72 | S96 | -32.1967 | 148.4975 | Native forests and woodlands | 6.10 | 2.638 | 43.834 | 16.614 | 0.011 | 0.001 | 626 | 17.3 |
| 73 | S97 | -32.007 | 147.9918 | Native forests and woodlands | 6.30 | 2.771 | 28.831 | 10.404 | 0.049 | 0.003 | 538 | 18 |
| 74 | S98 | -32.007 | 147.9918 | Native forests and woodlands | 5.64 | 4.128 | 40.983 | 9.928 | 0.116 | 0.007 | 538 | 18 |
| 75 | S99 | -32.007 | 147.9918 | Native forests and woodlands | 6.04 | 3.336 | 37.410 | 11.215 | 0.044 | 0.003 | 538 | 18 |
| 76 | S100 | -31.7 | 147.4667 | Native shrublands | 6.04 | 3.242 | 39.429 | 12.161 | 0.064 | 0.009 | 487 | 18.3 |
| 77 | S101 | -31.7 | 147.4667 | Native shrublands | 6.57 | 2.484 | 29.431 | 11.849 | 0.042 | 0.008 | 487 | 18.3 |
| 78 | S102 | -31.7 | 147.4667 | Native shrublands | 6.85 | 2.238 | 25.627 | 11.450 | 0.040 | 0.001 | 487 | 18.3 |
| 79 | S103 | -31.5466 | 146.7617 | Native forests and woodlands | 6.95 | 3.770 | 61.001 | 16.180 | 0.046 | 0.002 | 456 | 18.4 |
| 80 | S104 | -31.5466 | 146.7617 | Native forests and woodlands | 6.41 | 2.377 | 36.081 | 15.179 | 0.029 | 0.007 | 456 | 18.4 |
| 81 | S105 | -31.5466 | 146.7617 | Native forests and woodlands | 6.93 | 4.603 | 69.592 | 15.118 | 0.038 | 0.008 | 456 | 18.4 |
| 82 | S110 | -28.0819 | 145.7509 | Native forests and woodlands | 8.12 | 0.779 | 8.540 | 10.959 | 0.002 | 0.001 | 387 | 20.5 |
| 83 | S111 | -28.0819 | 145.7509 | Native forests and woodlands | 6.99 | 0.666 | 7.871 | 11.817 | 0.003 | 0.002 | 387 | 20.5 |
| 84 | S112 | -28.0819 | 145.7509 | Native forests and woodlands | 7.18 | 0.536 | 5.627 | 10.506 | 0.002 | 0.001 | 387 | 20.5 |
| 85 | S113 | -28.1378 | 144.8638 | Native shrublands | 7.02 | 0.633 | 6.137 | 9.697 | 0.003 | 0.001 | 361 | 20.5 |
| 86 | S114 | -28.1378 | 144.8638 | Native shrublands | 6.47 | 0.953 | 10.447 | 10.966 | 0.004 | 0.001 | 361 | 20.5 |
| 87 | S115 | -28.1378 | 144.8638 | Native shrublands | 5.87 | 0.615 | 5.828 | 9.475 | 0.004 | 0.001 | 361 | 20.5 |
| 88 | S116 | -27.9833 | 143.8167 | Native grasslands | 6.12 | 0.433 | 4.049 | 9.358 | 0.003 | 0.001 | 299 | 21.7 |
| 89 | S117 | -27.9833 | 143.8167 | Native grasslands | 6.22 | 0.445 | 4.086 | 9.183 | 0.003 | 0.001 | 299 | 21.7 |
| 90 | S118 | -27.9833 | 143.8167 | Native grasslands | 6.18 | 0.459 | 4.719 | 10.287 | 0.001 | 0.002 | 299 | 21.7 |
| 91 | S119 | -27.3872 | 144.3697 | Native shrublands | 7.44 | 1.788 | 22.839 | 12.773 | 0.020 | 0.001 | 340 | 21.4 |
| 92 | S120 | -27.3872 | 144.3697 | Native shrublands | 7.14 | 1.677 | 20.453 | 12.200 | 0.045 | 0.003 | 340 | 21.4 |
| 93 | S121 | -27.3872 | 144.3697 | Native shrublands | 6.44 | 1.949 | 23.976 | 12.302 | 0.033 | 0.002 | 340 | 21.4 |
| 94 | S123 | -26.5865 | 144.3028 | Native shrublands | 6.94 | 0.564 | 4.859 | 8.620 | 0.006 | 0.001 | 354 | 22.1 |
| 95 | S124 | -26.5865 | 144.3028 | Native shrublands | 6.31 | 0.814 | 8.083 | 9.927 | 0.008 | 0.001 | 354 | 22.1 |
| 96 | S125 | -26.5865 | 144.3028 | Native shrublands | 6.53 | 0.628 | 5.572 | 8.879 | 0.006 | 0.001 | 354 | 22.1 |
| 97 | S126 | -25.3667 | 142.75 | Native grasslands | 5.95 | 0.401 | 4.113 | 10.252 | 0.001 | 0.001 | 287 | 23 |
| 98 | S127 | -25.3667 | 142.75 | Native grasslands | 6.11 | 0.360 | 3.676 | 10.203 | 0.003 | 0.002 | 287 | 23 |
| 99 | S128 | -25.3667 | 142.75 | Native grasslands | 6.25 | 0.347 | 3.185 | 9.192 | 0.001 | 0.002 | 287 | 23 |
| 100 | S129 | -23.5 | 144.2333 | Native grasslands | 6.35 | 0.832 | 9.110 | 10.953 | 0.006 | 0.001 | 440 | 23.3 |
| 101 | S130 | -23.5 | 144.2333 | Native grasslands | 6.47 | 0.843 | 8.811 | 10.450 | 0.004 | 0.001 | 440 | 23.3 |
| 102 | S131 | -23.5 | 144.2333 | Native grasslands | 7.02 | 0.773 | 8.195 | 10.596 | 0.009 | 0.005 | 440 | 23.3 |
| 103 | S136 | -22.3861 | 143.1281 | Native grasslands | 7.81 | 0.443 | 4.314 | 9.743 | 0.001 | 0.001 | 416 | 24 |
| 104 | S137 | -22.3861 | 143.1281 | Native grasslands | 7.94 | 0.643 | 7.206 | 11.210 | 0.001 | 0.002 | 416 | 24 |
| 105 | S138 | -22.3861 | 143.1281 | Native grasslands | 7.79 | 0.567 | 5.811 | 10.241 | 0.001 | 0.001 | 416 | 24 |
| 106 | S139 | -22.97 | 139.88 | Native grasslands | 8.30 | 0.193 | 2.985 | 15.492 | 0.003 | 0.001 | 272 | 24.3 |
| 107 | S140 | -22.97 | 139.88 | Native grasslands | 7.85 | 0.199 | 2.450 | 12.288 | 0.001 | 0.002 | 272 | 24.3 |
| 108 | S141 | -22.97 | 139.88 | Native grasslands | 7.72 | 0.342 | 4.690 | 13.729 | 0.002 | 0.001 | 272 | 24.3 |
| 109 | S149 | -23.02 | 134.9 | Native shrublands | 6.48 | 1.877 | 22.379 | 11.925 | 0.014 | 0.009 | 333 | 20.5 |
| 110 | S150 | -23.02 | 134.9 | Native shrublands | 6.28 | 1.242 | 15.517 | 12.490 | 0.003 | 0.005 | 333 | 20.5 |
| 111 | S151 | -23.02 | 134.9 | Native shrublands | 6.60 | 1.096 | 13.622 | 12.433 | 0.004 | 0.006 | 333 | 20.5 |
| 112 | S156 | -23.02 | 134.41 | Native shrublands | 7.17 | 2.305 | 32.163 | 13.957 | 0.035 | 0.006 | 322 | 20.8 |
| 113 | S157 | -23.02 | 134.41 | Native shrublands | 8.43 | 1.154 | 14.952 | 12.953 | 0.022 | 0.007 | 322 | 20.8 |
| 114 | S158 | -23.02 | 134.41 | Native shrublands | 6.30 | 3.055 | 37.798 | 12.374 | 0.079 | 0.007 | 322 | 20.8 |
| 115 | S174 | -20.05 | 134.2 | Native shrublands | 6.12 | 0.689 | 8.665 | 12.574 | 0.012 | 0.008 | 385 | 25 |
| 116 | S175 | -20.05 | 134.2 | Native shrublands | 6.16 | 0.748 | 9.027 | 12.065 | 0.021 | 0.007 | 385 | 25 |
| 117 | S176 | -20.05 | 134.2 | Native shrublands | 6.14 | 0.543 | 6.184 | 11.400 | 0.008 | 0.007 | 385 | 25 |
| 118 | S180 | -19.8333 | 136 | Native shrublands | 7.01 | 0.610 | 6.415 | 10.522 | 0.003 | 0.009 | 351 | 25.4 |
| 119 | S181 | -19.8333 | 136 | Native shrublands | 6.97 | 0.555 | 5.395 | 9.715 | 0.003 | 0.007 | 351 | 25.4 |
| 120 | S182 | -19.8333 | 136 | Native shrublands | 6.94 | 0.718 | 8.579 | 11.945 | 0.004 | 0.009 | 351 | 25.4 |
| 121 | S189 | -20.35 | 139.1333 | Native shrublands | 5.00 | 0.815 | 10.749 | 13.194 | 0.025 | 0.006 | 414 | 24.5 |
| 122 | S190 | -20.35 | 139.1333 | Native shrublands | 4.93 | 0.724 | 9.989 | 13.790 | 0.017 | 0.005 | 414 | 24.5 |
| 123 | S191 | -20.35 | 139.1333 | Native shrublands | 5.21 | 0.614 | 9.213 | 15.000 | 0.017 | 0.002 | 414 | 24.5 |
| 124 | S192 | -20.33 | 140.25 | Native shrublands | 6.17 | 0.729 | 7.303 | 10.024 | 0.008 | 0.001 | 495 | 25.5 |
| 125 | S193 | -20.33 | 140.25 | Native shrublands | 6.39 | 0.600 | 6.033 | 10.056 | 0.003 | 0.001 | 495 | 25.5 |
| 126 | S194 | -20.33 | 140.25 | Native shrublands | 6.34 | 0.581 | 4.999 | 8.601 | 0.003 | 0.002 | 495 | 25.5 |
| 127 | S195 | -19.8903 | 140.2082 | Native shrublands | 7.32 | 0.426 | 5.879 | 13.795 | 0.001 | 0.002 | 514 | 25.7 |
| 128 | S196 | -19.8903 | 140.2082 | Native shrublands | 7.36 | 0.293 | 2.901 | 9.899 | 0.001 | 0.001 | 514 | 25.7 |
| 129 | S197 | -19.8903 | 140.2082 | Native shrublands | 6.91 | 0.360 | 3.981 | 11.055 | 0.001 | 0.001 | 514 | 25.7 |
| 130 | S198 | -19.9401 | 141.0762 | Native shrublands | 6.63 | 0.787 | 9.489 | 12.059 | 0.004 | 0.002 | 510 | 26 |
| 131 | S199 | -19.9401 | 141.0762 | Native shrublands | 6.66 | 0.893 | 12.082 | 13.531 | 0.004 | 0.002 | 510 | 26 |
| 132 | S200 | -19.9401 | 141.0762 | Native shrublands | 6.64 | 1.025 | 12.948 | 12.629 | 0.003 | 0.003 | 510 | 26 |
| 133 | S205 | -19.2833 | 145.5667 | Native forests and woodlands | 5.48 | 0.983 | 13.926 | 14.170 | 0.002 | 0.007 | 648 | 22.7 |
| 134 | S206 | -19.2833 | 145.5667 | Native forests and woodlands | 5.45 | 1.119 | 16.004 | 14.298 | 0.002 | 0.007 | 648 | 22.7 |
| 135 | S207 | -19.2833 | 145.5667 | Native forests and woodlands | 7.12 | 1.394 | 20.723 | 14.864 | 0.001 | 0.009 | 648 | 22.7 |
| 136 | S212 | -19.62466 | 147.47626 | Native forests and woodlands | 6.48 | 1.363 | 15.946 | 11.701 | 0.002 | 0.003 | 1060 | 23.9 |
| 137 | S213 | -19.62466 | 147.47626 | Native forests and woodlands | 6.19 | 1.881 | 24.460 | 13.005 | 0.001 | 0.005 | 1060 | 23.9 |
| 138 | S214 | -19.62466 | 147.47626 | Native forests and woodlands | 6.44 | 1.523 | 18.776 | 12.329 | 0.001 | 0.003 | 1060 | 23.9 |
| 139 | S215 | -20.2835 | 148.5364 | Native shrublands | 5.71 | 2.713 | 35.177 | 12.967 | 0.001 | 0.022 | 1271 | 23.2 |
| 140 | S216 | -20.2835 | 148.5364 | Native shrublands | 5.67 | 2.899 | 36.774 | 12.685 | 0.009 | 0.021 | 1271 | 23.2 |
| 141 | S217 | -20.2835 | 148.5364 | Native shrublands | 5.60 | 2.852 | 38.028 | 13.333 | 0.012 | 0.028 | 1271 | 23.2 |
| 142 | S218 | -21.14389 | 149.07854 | Native forests and woodlands | 5.19 | 1.447 | 16.865 | 11.655 | 0.005 | 0.006 | 1758 | 22.5 |
| 143 | S219 | -21.14389 | 149.07854 | Native forests and woodlands | 5.59 | 1.736 | 20.484 | 11.801 | 0.005 | 0.006 | 1758 | 22.5 |
| 144 | S220 | -21.14389 | 149.07854 | Native forests and woodlands | 5.20 | 1.472 | 16.075 | 10.918 | 0.004 | 0.007 | 1758 | 22.5 |
| 145 | S221 | -22.0333 | 149.4667 | Native forests and woodlands | 6.39 | 2.727 | 38.398 | 14.080 | 0.002 | 0.035 | 971 | 21.4 |
| 146 | S222 | -22.0333 | 149.4667 | Native forests and woodlands | 6.31 | 1.805 | 24.746 | 13.714 | 0.001 | 0.025 | 971 | 21.4 |
| 147 | S223 | -22.0333 | 149.4667 | Native forests and woodlands | 6.44 | 2.662 | 35.211 | 13.230 | 0.001 | 0.037 | 971 | 21.4 |
| 148 | S225 | -22.8333 | 150.6667 | Plantation forests | 6.68 | 2.293 | 38.388 | 16.741 | 0.001 | 0.002 | 1148 | 22.3 |
| 149 | S226 | -22.8333 | 150.6667 | Plantation forests | 6.58 | 1.970 | 31.950 | 16.215 | 0.001 | 0.001 | 1148 | 22.3 |
| 150 | S227 | -22.8333 | 150.6667 | Plantation forests | 6.66 | 2.113 | 34.783 | 16.458 | 0.001 | 0.002 | 1148 | 22.3 |
| 151 | S228 | -23.6734 | 150.9761 | Native forests and woodlands | 8.31 | 1.524 | 30.021 | 19.703 | 0.001 | 0.001 | 821 | 22.3 |
| 152 | S229 | -23.6734 | 150.9761 | Native forests and woodlands | 8.47 | 0.909 | 19.246 | 21.172 | 0.001 | 0.003 | 821 | 22.3 |
| 153 | S230 | -23.6734 | 150.9761 | Native forests and woodlands | 8.57 | 0.837 | 20.007 | 23.892 | 0.001 | 0.001 | 821 | 22.3 |
| 154 | S231 | -24.4167 | 151.55 | Native forests and woodlands | 7.22 | 1.364 | 21.794 | 15.983 | 0.005 | 0.004 | 1094 | 21.6 |
| 155 | S232 | -24.4167 | 151.55 | Native forests and woodlands | 6.30 | 1.539 | 18.330 | 11.908 | 0.004 | 0.004 | 1094 | 21.6 |
| 156 | S233 | -24.4167 | 151.55 | Native forests and woodlands | 6.42 | 1.426 | 25.104 | 17.610 | 0.001 | 0.001 | 1094 | 21.6 |
| 157 | S235 | -25.3 | 152.4667 | Native forests and woodlands | 6.05 | 0.810 | 10.630 | 13.129 | 0.016 | 0.004 | 1082 | 21.4 |
| 158 | S236 | -25.3 | 152.4667 | Native forests and woodlands | 6.75 | 0.880 | 11.455 | 13.011 | 0.035 | 0.002 | 1082 | 21.4 |
| 159 | S237 | -25.3 | 152.4667 | Native forests and woodlands | 6.04 | 0.864 | 12.121 | 14.031 | 0.015 | 0.002 | 1082 | 21.4 |
| 160 | S238 | -25.7611 | 152.9339 | Native forests and woodlands | 6.36 | 0.392 | 6.934 | 17.671 | 0.001 | 0.003 | 1286 | 21.3 |
| 161 | S239 | -25.7611 | 152.9339 | Native forests and woodlands | 6.34 | 0.588 | 11.787 | 20.049 | 0.001 | 0.002 | 1286 | 21.3 |
| 162 | S240 | -25.7611 | 152.9339 | Native forests and woodlands | 6.14 | 0.482 | 8.743 | 18.147 | 0.001 | 0.005 | 1286 | 21.3 |
| 163 | S241 | -26.9139 | 153.0546 | Plantation forests | 5.98 | 0.855 | 14.642 | 17.130 | 0.001 | 0.004 | 1566 | 20.3 |
| 164 | S242 | -26.9139 | 153.0546 | Plantation forests | 6.00 | 0.790 | 12.935 | 16.366 | 0.002 | 0.004 | 1566 | 20.3 |
| 165 | S243 | -26.9139 | 153.0546 | Plantation forests | 5.89 | 0.782 | 13.296 | 16.993 | 0.001 | 0.008 | 1566 | 20.3 |
| 166 | S244 | -27.5889 | 153.1656 | Native forests and woodlands | 5.60 | 2.120 | 32.342 | 15.256 | 0.001 | 0.003 | 1192 | 20.1 |
| 167 | S245 | -27.5889 | 153.1656 | Native forests and woodlands | 5.45 | 2.387 | 36.558 | 15.316 | 0.001 | 0.005 | 1192 | 20.1 |
| 168 | S246 | -27.5889 | 153.1656 | Native forests and woodlands | 4.98 | 2.133 | 32.144 | 15.069 | 0.001 | 0.005 | 1192 | 20.1 |
| 169 | S247 | -28.6442 | 153.6216 | Native forests and woodlands | 4.97 | 1.889 | 39.032 | 20.668 | 0.001 | 0.002 | 1816 | 19.7 |
| 170 | S248 | -28.6442 | 153.6216 | Native forests and woodlands | 5.26 | 1.148 | 25.017 | 21.786 | 0.001 | 0.001 | 1816 | 19.7 |
| 171 | S249 | -28.6442 | 153.6216 | Native forests and woodlands | 5.29 | 1.719 | 35.870 | 20.862 | 0.001 | 0.002 | 1816 | 19.7 |
| 172 | S250 | -29.1788 | 153.2604 | Native forests and woodlands | 5.60 | 1.437 | 22.562 | 15.698 | 0.001 | 0.004 | 1407 | 19.2 |
| 173 | S251 | -29.1788 | 153.2604 | Native forests and woodlands | 5.71 | 1.401 | 21.416 | 15.290 | 0.001 | 0.004 | 1407 | 19.2 |
| 174 | S252 | -29.1788 | 153.2604 | Native forests and woodlands | 6.00 | 1.403 | 21.101 | 15.041 | 0.001 | 0.006 | 1407 | 19.2 |
| 175 | S253 | -30.1888 | 153.146 | Native forests and woodlands | 6.13 | 3.222 | 65.843 | 20.437 | 0.001 | 0.007 | 1790 | 18.3 |
| 176 | S254 | -30.1888 | 153.146 | Native forests and woodlands | 5.93 | 2.928 | 54.495 | 18.614 | 0.001 | 0.011 | 1790 | 18.3 |
| 177 | S255 | -30.1888 | 153.146 | Native forests and woodlands | 6.82 | 3.507 | 70.790 | 20.185 | 0.001 | 0.016 | 1790 | 18.3 |
| 178 | S256 | -31.2634 | 152.8173 | Native forests and woodlands | 5.59 | 1.496 | 27.071 | 18.099 | 0.001 | 0.006 | 1367 | 17.8 |
| 179 | S257 | -31.2634 | 152.8173 | Native forests and woodlands | 5.56 | 1.442 | 29.448 | 20.427 | 0.001 | 0.006 | 1367 | 17.8 |
| 180 | S258 | -31.2634 | 152.8173 | Native forests and woodlands | 5.56 | 0.998 | 22.043 | 22.092 | 0.001 | 0.003 | 1367 | 17.8 |
| 181 | S259 | -31.9514 | 152.4256 | Native forests and woodlands | 5.70 | 3.398 | 58.580 | 17.242 | 0.001 | 0.007 | 1239 | 17.9 |
| 182 | S260 | -31.9514 | 152.4256 | Native forests and woodlands | 5.71 | 4.073 | 68.681 | 16.864 | 0.001 | 0.007 | 1239 | 17.9 |
| 183 | S261 | -31.9514 | 152.4256 | Native forests and woodlands | 5.64 | 4.023 | 69.152 | 17.188 | 0.001 | 0.007 | 1239 | 17.9 |
| 184 | S262 | -32.82402 | 151.85085 | Native forests and woodlands | 5.51 | 2.944 | 48.167 | 16.363 | 0.006 | 0.003 | 1175 | 17.9 |
| 185 | S263 | -32.82402 | 151.85085 | Native forests and woodlands | 5.75 | 1.384 | 21.794 | 15.749 | 0.001 | 0.001 | 1175 | 17.9 |
| 186 | S264 | -32.82402 | 151.85085 | Native forests and woodlands | 5.71 | 1.673 | 25.701 | 15.366 | 0.001 | 0.003 | 1175 | 17.9 |
| 187 | S265 | -33.525 | 151.0878 | Native forests and woodlands | 5.80 | 1.463 | 22.361 | 15.283 | 0.005 | 0.006 | 1175 | 16.7 |
| 188 | S266 | -33.525 | 151.0878 | Native forests and woodlands | 5.84 | 1.742 | 26.566 | 15.250 | 0.006 | 0.005 | 1175 | 16.7 |
| 189 | S267 | -33.525 | 151.0878 | Native forests and woodlands | 5.96 | 1.364 | 21.274 | 15.601 | 0.001 | 0.007 | 1175 | 16.7 |
| 190 | S268 | -34.1318 | 151.1178 | Native shrublands | 5.88 | 0.957 | 27.315 | 28.548 | 0.001 | 0.003 | 1298 | 17.1 |
| 191 | S269 | -34.1318 | 151.1178 | Native shrublands | 5.88 | 1.233 | 36.301 | 29.441 | 0.001 | 0.002 | 1298 | 17.1 |
| 192 | S270 | -34.1318 | 151.1178 | Native shrublands | 5.41 | 2.208 | 59.275 | 26.847 | 0.001 | 0.013 | 1298 | 17.1 |
| 193 | S271 | -34.4589 | 150.514 | Native forests and woodlands | 5.75 | 3.163 | 76.167 | 24.078 | 0.003 | 0.009 | 1273 | 14.1 |
| 194 | S272 | -34.4589 | 150.514 | Native forests and woodlands | 6.15 | 1.669 | 40.754 | 24.420 | 0.001 | 0.004 | 1273 | 14.1 |
| 195 | S273 | -34.4589 | 150.514 | Native forests and woodlands | 6.32 | 1.795 | 42.059 | 23.438 | 0.001 | 0.003 | 1273 | 14.1 |
| 196 | S275 | -34.8133 | 148.6314 | Native forests and woodlands | 5.32 | 1.433 | 23.044 | 16.077 | 0.005 | 0.001 | 741 | 13.9 |
| 197 | S276 | -34.8133 | 148.6314 | Native forests and woodlands | 5.45 | 1.391 | 19.830 | 14.252 | 0.005 | 0.001 | 741 | 13.9 |
| 198 | S277 | -34.8133 | 148.6314 | Native forests and woodlands | 5.30 | 1.185 | 18.629 | 15.718 | 0.005 | 0.004 | 741 | 13.9 |
| 199 | S278 | -35.2269 | 147.77889 | Native forests and woodlands | 5.78 | 1.686 | 18.120 | 10.745 | 0.005 | 0.001 | 714 | 15 |
| 200 | S279 | -35.2269 | 147.77889 | Native forests and woodlands | 5.57 | 1.294 | 16.114 | 12.450 | 0.015 | 0.002 | 714 | 15 |
| 201 | S280 | -35.2269 | 147.77889 | Native forests and woodlands | 6.14 | 1.054 | 13.691 | 12.984 | 0.002 | 0.002 | 714 | 15 |
| 202 | S281 | -35.84188 | 147.20339 | Native forests and woodlands | 5.37 | 3.321 | 59.603 | 17.948 | 0.002 | 0.003 | 849 | 13.7 |
| 203 | S282 | -35.84188 | 147.20339 | Native forests and woodlands | 5.31 | 3.630 | 66.069 | 18.200 | 0.003 | 0.021 | 849 | 13.7 |
| 204 | S283 | -35.84188 | 147.20339 | Native forests and woodlands | 5.51 | 1.277 | 15.010 | 11.750 | 0.004 | 0.002 | 849 | 13.7 |
| 205 | S284 | -36.10217 | 146.69762 | Native grasslands | 5.33 | 1.688 | 20.253 | 12.001 | 0.010 | 0.049 | 700 | 14.7 |
| 206 | S285 | -36.10217 | 146.69762 | Native grasslands | 5.44 | 1.167 | 15.775 | 13.521 | 0.008 | 0.005 | 700 | 14.7 |
| 207 | S286 | -36.10217 | 146.69762 | Native grasslands | 5.09 | 4.289 | 72.733 | 16.958 | 0.007 | 0.001 | 700 | 14.7 |
| 208 | S287 | -36.50609 | 146.09569 | Native grasslands | 5.14 | 4.266 | 69.089 | 16.194 | 0.014 | 0.001 | 709 | 14.4 |
| 209 | S288 | -36.50609 | 146.09569 | Native grasslands | 4.64 | 4.359 | 75.920 | 17.416 | 0.020 | 0.012 | 709 | 14.4 |
| 210 | S289 | -36.50609 | 146.09569 | Native grasslands | 5.01 | 1.668 | 22.648 | 13.577 | 0.016 | 0.031 | 709 | 14.4 |
| 211 | S290 | -36.77763 | 145.55906 | Native forests and woodlands | 5.11 | 1.184 | 15.033 | 12.701 | 0.002 | 0.008 | 698 | 14.4 |
| 212 | S291 | -36.77763 | 145.55906 | Native forests and woodlands | 5.25 | 1.281 | 16.076 | 12.551 | 0.004 | 0.001 | 698 | 14.4 |
| 213 | S292 | -36.77763 | 145.55906 | Native forests and woodlands | 5.83 | 1.631 | 24.314 | 14.910 | 0.003 | 0.013 | 698 | 14.4 |
| 214 | S293 | -37.21946 | 145.03143 | Native grasslands | 6.15 | 1.590 | 24.101 | 15.155 | 0.004 | 0.002 | 803 | 13.2 |
| 215 | S294 | -37.21946 | 145.03143 | Native grasslands | 6.24 | 4.182 | 62.360 | 14.911 | 0.003 | 0.001 | 803 | 13.2 |
| 216 | S295 | -37.21946 | 145.03143 | Native grasslands | 5.52 | 1.850 | 17.589 | 9.505 | 0.036 | 0.001 | 803 | 13.2 |

# **Table S2**. The percentage of cross-group interactions between functional groups of protists and other soil organisms

| Interactions | Protists – Bacteria | Protists – Fungi | Protists – Invertebrates | Average cross-group interactions |
| --- | --- | --- | --- | --- |
| Number of cross-group interactions | 326 | 9 | 27 | - |
| Consumers (%) | 50.61 | 44.44 | 51.85 | 48.97 |
| Phototrophs (%) | 33.74 | 33.33 | 22.22 | 29.77 |
| Parasites (%) | 2.76 | 22.22 | 11.11 | 12.03 |
| Unknown (%) | 12.88 | 0.00 | 14.81 | 9.23 |
